# Determinants of Uncontrolled Hypertension in Rural Communities in South Asia—Bangladesh, Pakistan, and Sri Lanka

**DOI:** 10.1093/ajh/hpy071

**Published:** 2018-04-26

**Authors:** Tazeen H Jafar, Mihir Gandhi, Imtiaz Jehan, Aliya Naheed, H Asita de Silva, Hunaina Shahab, Dewan Alam, Nathasha Luke, Ching Wee Lim, COBRA-BPS Study Group

**Affiliations:** 1 Program in Health Services & Systems Research, Duke-NUS Medical School, Singapore; 2 Duke Global Health Institute, Duke University, Durham, North Carolina, USA; 3 Biostatistics, Singapore Clinical Research Institute, Singapore; 4 Centre for Quantitative Medicine, Duke-NUS Medical School, Singapore; 5 Tampere Center for Child Health Research, University of Tampere and Tampere University Hospital, Tampere, Finland; 6 Department of Community Health Sciences, Aga Khan University, Karachi, Pakistan; 7 International Centre for Diarrhoeal Disease Research, Dhaka, Bangladesh; 8 Clinical Trials Unit, Department of Pharmacology, Faculty of Medicine, University of Kelaniya, Kelaniya, Sri Lanka; 9 School of Kinesiology and Health Science, Faculty of Health, York University, Toronto, Ontario, Canada

**Keywords:** adherence, antihypertensive medications, blood pressure, cardiovascular risk, hypertension

## Abstract

**BACKGROUND:**

Uncontrolled blood pressure (BP) is a leading risk factor for death and disability in South Asia. We aimed to determine the cross-country variation, and the factors associated with uncontrolled BP among adults treated for hypertension in rural South Asia.

**METHODS:**

We enrolled 1,718 individuals aged ≥40 years treated for hypertension in a cross-sectional study from rural communities in Bangladesh, Pakistan, and Sri Lanka. Multivariable logistic regression model was used to determine the factors associated with uncontrolled BP (systolic BP ≥140 mmHg or diastolic BP ≥90 mmHg).

**RESULTS:**

Among hypertensive individuals, 58.0% (95% confidence interval (CI) 55.7, 60.4) had uncontrolled BP: 52.8% (49.0, 56.6) in Bangladesh, 70.6% (65.7, 75.1) in Pakistan, and 56.5% (52.7, 60.1) in Sri Lanka. The odds (odds ratio (95% CI)) of uncontrolled BP were significantly higher in individuals with lower wealth index (1.17 (1.02, 1.35)); single vs. married (1.46 (1.10, 1.93)); higher log urine albumin-to-creatinine ratio (1.41 (1.24, 1.60)); lower estimated glomerular filtration rate (1.23 (1.01, 1.49)); low vs. high adherence to antihypertensive medication (1.50 (1.16, 1.94)); and Pakistan (2.91 (1.60, 5.28)) vs. Sri Lanka. However, the odds were lower in those with vs. without self-reported kidney disease (0.51 (0.28, 0.91)); and receiving vs. not receiving statins (0.62 (0.44, 0.87)).

**CONCLUSIONS:**

The majority of individuals with treated hypertension have uncontrolled BP in rural Bangladesh, Pakistan, and Sri Lanka with significant disparities among and within countries. Urgent public health efforts are needed to improve access and adherence to antihypertensive medications in disadvantaged populations in rural South Asia.

Hypertension is a leading attributable risk factor for cardiovascular disease (CVD) and kidney disease, and premature death globally.^1^ Evidence from epidemiological studies and clinical trials have clearly demonstrated that the risk of blood pressure (BP) with adverse CVD outcomes is continuous, and more pronounced at higher levels of BP. Fortunately, lowering BP reduces this risk. Currently, over 1.1 billion people are affected with hypertension globally, and this number is expected to increase to more than 1.5 billion by 2025.^2^ The rise in the number of people with hypertension is projected to be particularly steep in South Asia where population has shown to be at high risk, trends in age-standardized BP levels over the past 4 decades show persistent rise, and the prevalence of hypertension is already 40%.^2–4^

Unfortunately, a significant proportion of the population with hypertension is unaware and untreated in South Asian countries. However, BP control rates are grossly inadequate (<30% controlled to <140/90 mm Hg) even among those diagnosed and treated for hypertension. Studies done elsewhere show several barriers to hypertension control at the individual, community, as well as health systems levels including lack of adherence to antihypertensive medications, and insufficient medication intensification^5–7^ Most of South Asia is still rural (73% Bangladesh, 64% Pakistan, 71% India, 85% Sri Lanka) where the population, healthcare infrastructure and healthcare provider characteristics are very different compared to the urban setting.^8^ Furthermore, the impact of poor BP control is expected to be much worse in rural areas where CVD case fatality rates have shown to be higher than in urban areas.^9^ However, there is scarcity of information on factors potentially responsible for uncontrolled BP among treated hypertensives in South Asia. This information is essential to focus national attention on comprehensive and effective public-health action on hypertension control strategies.

We analyzed data on 1,718 individuals aged 40 years or older treated for hypertension from rural communities of Bangladesh, Pakistan, and Sri Lanka with the following objectives:

(1) To determine the cross-country variation in the proportion of individuals with uncontrolled BP among adults with treated hypertension;
(2) To evaluate the association between the sociodemographic factors, lifestyle, comorbid conditions, and adherence to antihypertensive medications with uncontrolled BP.

We hypothesized that: (i) there will be cross-country variation in the proportion of individuals with uncontrolled BP; (ii) the cross-country variation in uncontrolled BP will not be fully accounted for by differences in lifestyle factors, comorbid conditions, or adherence to antihypertensive medications.

## METHODS

### Study design and sampling

This was a cross-sectional study conducted as a part of baseline assessments in the ongoing cluster-randomized trial, Control of Blood Pressure and Risk Attenuation—rural Bangladesh, Pakistan, Sri Lanka (COBRA-BPS), in 30 randomly selected communities rural Bangladesh, Pakistan, and Sri Lanka.

The study was conducted in Tangail and Munshigaonj districts (population 3.2 million) of Bangladesh; Thatta district (population 1.5 million) of Pakistan, and Putlam district (population 0.7 million) of Sri Lanka. A cluster was defined by 250–300 households as listed in local administration sampling frames. These clusters are grouped in geographically contiguous administrative units of 10–15 clusters such that each unit is served by a government clinic. For the random selection of clusters, a total of 30 administrative units were randomly selected from the 3 countries (10 in each) followed by a random selection of 1 cluster per unit, stratified by distance from the government clinic in each administrative unit thereby ensuring adequate representation of clusters located far from the clinic. More details regarding the trial design have been published previously.^10^

The study was approved by the local ethics review committees in each of 3 countries, UK and Singapore.

### Study participants and screening

The eligibility criteria for COBRA-BPS were age 40 years or more, residing in the selected clusters, and having hypertension as defined by either persistently elevated BP (systolic BP ≥140 mmHg or diastolic BP ≥90 mmHg) based on the mean of the last 2 of 3 measurements on 2 separate days, or on currently on antihypertensive medications.

Individuals who were permanently bed-ridden or too ill or with advanced medical disease (on dialysis, liver failure, other systemic disease), pregnant, or mentally compromised, and unable to give informed consent were considered ineligible for the study.

All BP assessors successfully completed training sessions on the use of the BP measurement protocol. Passing requirements for trained BP assessor consisted of satisfactory performance on a skills test assessing preparation of study participants for BP measurement, selection of an appropriate cuff size, standard BP measurement techniques, and concordant live BP measurements with an instructor using a calibrated automated “Omron HEM-7300 Blood Pressure Monitor,” which has been validated and recommended by the Association for the Advancement of Medical Instrumentation (AAMI), the British Hypertension Society (BHS), and the European Society of Hypertension.^11^

Adults aged 40 years or older residing in households in randomly selected clusters were approached to participate in the study (maximum 3 times if not available for any reasons). Informed consent was obtained before any assessments. Those already on antihypertensive medications were considered to have confirmed “treated hypertension” status, and enrolled in the study. More than 1 participants per household were enrolled if eligible. Participants were advised to refrain from coffee, or tea, cigarette smoking, and vigorous exercise for at least 30 minutes prior to their BP assessments.^10^ Three readings of BP were taken, each initiated by research staff 3 minutes apart using the Omron device. Uncontrolled hypertension was defined as systolic BP ≥140 mmHg or diastolic BP ≥90 mmHg based on last 2 of 3 readings among individuals receiving antihypertensive medications. The individuals not treated with antihypertensive medication with elevated BP (systolic BP ≥140 mmHg or diastolic BP ≥90 mmHg) were visited again after at least 1 week for re-measurement of BP for confirmation of newly diagnosed hypertension status. This study primarily reports findings on individuals with treated hypertension.

### Assessment and outcomes

Uncontrolled BP was defined as systolic BP ≥140 mmHg or diastolic BP ≥90 mmHg, and receiving antihypertensive medication. The definition was based on persistent elevated measurements obtained from the last 2 of 3 readings.

Information on sociodemographic, anthropometry, diet, lifestyle and behavior, comorbidities, use and adherence to antihypertensive medications were obtained on enrollment. Data collection forms were translated in local languages (Bengla, Urdu, Sindhi, Sinhala, and Tamil), that have been piloted in a feasibility study.^12^

The sociodemographic information included age, gender, education status (no formal education, received formal education), marital status defined as single (never married/separated/widowed) or married, and socioeconomic status as measured by the international wealth index. The wealth index was derived using weights proposed by Smits and Steendijk.^13^ It is a weighted sum of indicator variables for available facilities in the house plus a constant term. Facilities include having television, refrigerator, phone, car, bicycle, cheap utensils (table, chair, water cooker, clock/watch, lamp, and radio), expensive utensils (deep freezer, air conditioner, motorbike, washing machine, and computer), quality of floor material, quality of toilet facility, number of sleeping rooms, accessibility to electricity, and quality of water source. Higher wealth index indicates a better socioeconomic status.

Body mass index (BMI) and waist circumference were measured for the anthropometric information. Participants were classified as obese/over-weight if they had BMI ≥ 23.5 kg/m^2^.^14,15^

Weekly fruit and vegetable intake were collected using an abbreviated food frequency questionnaire.^12^ Physical activity level was assessed using the International Physical Activity questionnaire.^16^ The comorbid conditions included self-reported heart disease, stroke, diabetes, and kidney disease. Fasting blood samples were collected for plasma glucose, serum creatinine, lipids (total cholesterol, high-density lipoprotein (HDL) cholesterol, low-density lipoprotein (LDL) cholesterol, triglycerides), estimated glomerular filtration rate (eGFR), a morning spot urine sample for sodium, albumin, and creatinine. Serum creatinine measurements were calibrated to isotope dilution mass spectrometry (IDMS) and all laboratories adhered to external international quality control procedures.^17^ Dietary sodium was measured by spot urine sodium to creatinine ratio and converted into 24-hour urine sodium estimates using the Kawasaki formula.^18^

Current use of antihypertensive and statin medications was collected. Adherence to antihypertensive medications was assessed by administering the 8-items Morisky Medication Adherence scale (MMAS-8).^19–22^ Participants with a score of less than 6 on the scale were considered to have a low level of medication adherence, while those with a score of 6–7 were considered to be moderately adherent, and a score of more than 7 denotes a high level of adherence.

### Statistical analysis

Proportion of participants with uncontrolled BP was estimated along with corresponding 95% confidence interval (CI) for each country and overall including all the 3 countries. Participant characteristics were presented using descriptive statistics and compared between the controlled and uncontrolled BP groups using the chi-squared test and 2-sample independent *t*-test for categorical and continuous variables, respectively. Association between each of the participant characteristics and uncontrolled BP was evaluated using a separate logistic mixed-effect model (basic model) adjusted for age, gender, and country to account for potential imbalances in the key demographic and geographic characteristics. The association between the participant characteristics and uncontrolled BP was then assessed including all the participant characteristics in the same logistic regression model simultaneously (multivariable model). The multivariable model was further evaluated with the addition of interaction terms between country and participant characteristics to assess country-specific impact. All continuous variables were included as z-score standardized for mean and standard deviation (SD) in the models for interpretation of corresponding odds ratios in terms of per 1 SD increase in the variables. All the models included cluster-specific random intercepts to account for within cluster correlation. A *P*-value less than 0.05 was considered statistically significant.

Sensitivity analysis were conducted to assess the proportion of individuals with uncontrolled BP among those with treated hypertension (i) in each country by distance of clusters to the government clinic; (ii) by self-reported chronic disease groups defined as self-reported heart diseases or stroke or kidney diseases; and (iii) by economic status with IWI as a categorical variable (poor: <15 percentile, Middle: 15–85th percentile, Rich: >85th percentile).

## RESULTS

A total of 11,510 individuals aged 40 years or older were approached for screening during April 2016 to March 2017: 4,442 in Bangladesh, 4,760 in Pakistan, 2,308 in Sri Lanka. Of these, 895 (20.1%), 894 (18.8%), and 854 (37.0%) were hypertensive (treated and newly diagnosed). Supplementary Appendix Figure 1 shows cumulative study enrollment from all 3 countries. Among those with hypertension (*n* = 2,643), 670 (74.9%), 361 (40.4%), and 687 (80.4%) were on treatment with antihypertensive medications in Bangladesh, Pakistan, and Sri Lanka, respectively (Table 1). Among those with treated hypertension (*n* = 1,718), 58.0% (95% CI 55.7–60.3) had uncontrolled BP: 52.8% (95% CI 49.0–56.6) in Bangladesh, 70.6% (95% CI 65.7–75.1) in Pakistan, and 56.5% (95% CI 52.7–60.1) in Sri Lanka.

**Table 1.** Sociodemographic and disease characteristics of those who took antihypertensive medications by hypertension control status

	All countries (*N* = 1,718)			
Characteristics	All (*N* = 1,718, 100%)	Uncontrolled hypertensive patients^a^ (*N* = 997, 58.0%)	Controlled hypertensive patients (*N* = 721, 42.0%)	*P* (uncontrolled vs. controlled)
Demographic characteristics				
Age (years), Mean (SD)	59.70 (11.51)	59.83 (11.45)	59.50 (11.61)	0.561
Male, *n* (%)	523 (30.4)	287 (28.8)	236 (32.7)	0.079
Education, *n* (%)				0.216
No formal education	591 (34.4)	355 (35.6)	236 (32.7)	
Formal education	1,127 (65.6)	642 (64.4)	485 (67.3)	
Currently working, *n* (%)	421 (24.5)	238 (23.9)	183 (25.4)	0.473
Married, *n* (%)	1,197 (69.7)	662 (66.4)	535 (74.2)	0.001
Anthropometric characteristics				
Obese/overweight (≥23.5 BMI), *n* (%)	1,014 (59.0)	610 (61.2)	404 (56.0)	0.044
BMI (kg/m^2^), Mean (SD)	25.02 (4.80)	25.24 (4.88)	24.71 (4.67)	0.028
Waist circumference (cm), Mean (SD)	88.68 (12.69)	89.21 (12.46)	87.95 (12.97)	0.044
Socioeconomic characteristics				
International wealth index, Mean (SD)	61.83 (20.25)	61.18 (20.49)	62.73 (19.88)	0.117
Life-style characteristics				
Currently smoking, *n* (%)	115 (6.7)	65 (6.5)	50 (6.9)	0.734
Physical activity level, *n* (%)				0.026
Inactive	458 (26.7)	290 (29.1)	168 (23.3)	
Minimally active	974 (56.7)	544 (54.6)	430 (59.6)	
Highly active	286 (16.6)	163 (16.3)	123 (17.1)	
Self-reported health characteristics				
Chronic diseases related to hypertension^b^, *n* (%)	499 (29.0)	250 (25.1)	249 (34.5)	<0.001
Heart disease, *n* (%)	280 (16.3)	139 (13.9)	141 (19.6)	0.002
Stroke, *n* (%)	244 (14.2)	128 (12.8)	116 (16.1)	0.057
Kidney disease, *n* (%)	64 (3.7)	33 (3.3)	31 (4.3)	0.285
Diabetes, *n* (%)	459 (26.7)	274 (27.5)	185 (25.7)	0.399
Morisky adherence level for antihypertensive medications, *n* (%)				<0.001
Low	709 (41.3)	451 (45.2)	258 (35.8)	
Medium	428 (24.9)	234 (23.5)	194 (26.9)	
High	581 (33.8)	312 (31.3)	269 (37.3)	
Number of antihypertensive medications, *n* (%)				0.167
1	1,152 (67.1)	685 (68.7)	467 (64.8)	
2	446 (26.0)	242 (24.3)	204 (28.3)	
≥3	120 (7.0)	70 (7.0)	50 (6.9)	
Currently on statins, *n* (%)	324 (18.9)	154 (15.4)	170 (23.6)	<0.001
Food-intake and laboratory measures				
Vegetables intake (at least once a week), *n* (%)	1,636 (95.2)	952 (95.5)	684 (94.9)	0.553
Fruits intake (at least once a week), *n* (%)	939 (54.7)	540 (54.2)	399 (55.3)	0.629
24-hour urine sodium (g/day), Mean (SD)	4.71 (1.51)	4.73 (1.53)	4.69 (1.49)	0.674
Log_e_ urine spot albumin-to-creatinine ratio (mg/g), Mean (SD)	2.79 (1.38)	2.95 (1.42)	2.56 (1.29)	<0.001
Systolic blood pressure (mmHg), Mean (SD)	143.81 (23.31)	158.53 (18.35)	123.46 (10.74)	<0.001
Diastolic blood pressure (mmHg), Mean (SD)	86.20 (14.80)	94.55 (12.34)	74.67 (9.05)	<0.001
Fasting plasma glucose (mg/dl), Mean (SD)	116.89 (46.42)	120.73 (52.34)	111.66 (36.25)	<0.001
eGFR (ml/minute per 1.73 m^2^), Mean (SD)	70.81 (24.60)	70.95 (24.67)	70.61 (24.52)	0.782
Total cholesterol (mg/dl), Mean (SD)	196.10 (47.48)	199.67 (48.66)	191.23 (45.41)	<0.001
HDL cholesterol (mg/dl), Mean (SD)	45.77 (12.97)	46.25 (13.02)	45.13 (12.89)	0.087
LDL cholesterol (mg/dl), Mean (SD)	125.43 (39.74)	127.57 (40.32)	122.53 (38.78)	0.012
Triglycerides (mg/dl), Mean (SD)	151.55 (92.18)	153.28 (91.94)	149.19 (92.53)	0.381

Abbreviations: BMI, body mass index; eGFR, estimated glomerular filtration rate; HDL, high-density lipoprotein; LDL, low-density lipoprotein; SD, standard deviation.

^a^Uncontrolled hypertension was defined as systolic blood pressure ≥140 mmHg or diastolic blood pressure ≥90 mmHg. Participant characteristics are defined in the methods section.

^b^Chronic disease related to hypertension were defined as self-reported presence of heart disease, stroke, or kidney disease.

**Figure 1.**
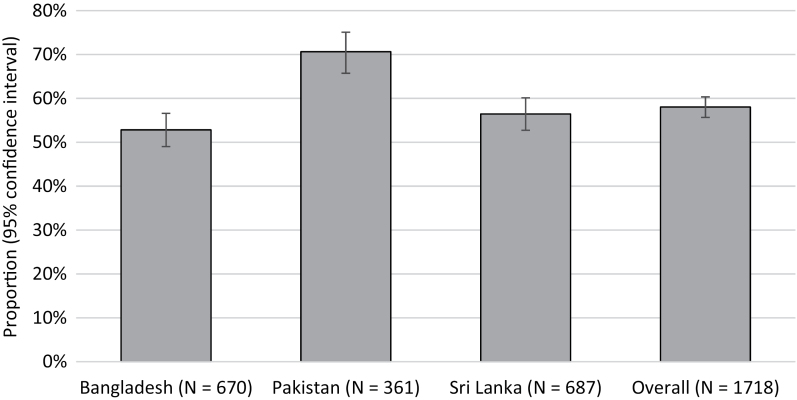
Proportion (95% confidence interval) of individuals with uncontrolled BP among those with treated hypertension.

**Figure 2.**
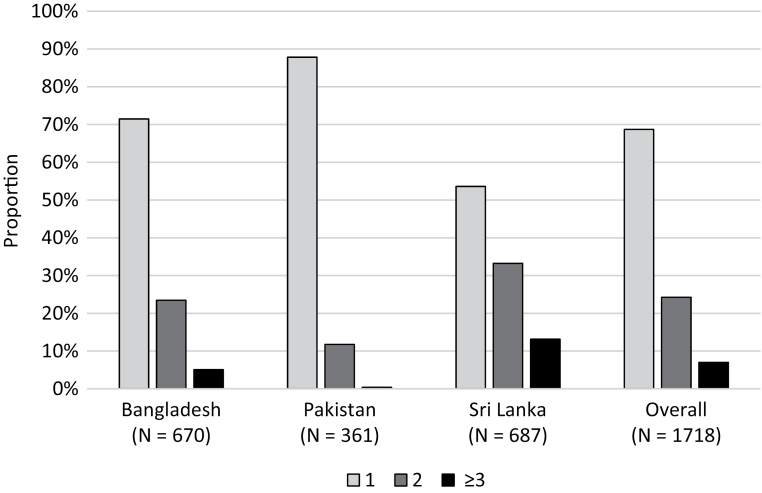
Proportion of individuals according to number of antihypertensive medications.

Figure 1 illustrates the proportion of individuals with uncontrolled BP. Of these, 69%, 24%, and 7% were receiving 1, 2, and 3 or more agents, respectively (for country-wise proportions, see Figure 2).

The comparison of sociodemographic, lifestyle, comorbid conditions, and adherence to antihypertensive medications between individuals with and without uncontrolled BP is shown in Table 1. Supplementary Appendix Table 1 shows the same comparison for stratified by country.

In the basic models (Table 2, column 2), lower wealth index (*P* = 0.045), being single vs. married (*P* = 0.007), low vs. high adherence to antihypertensive medications (*P* <0.001), not receiving vs. receiving statins (*P* <0.001), without vs. with concomitant self-reported heart disease (*P* = 0.010), higher urine albumin excretion (*P* < 0.001), higher fasting glucose (*P* < 0.001), lower eGFR (*P* = 0.040), and higher serum LDL cholesterol (*P =* 0.001) were associated with statistically significantly higher odds of uncontrolled BP. Pakistan had significantly higher odds of uncontrolled BP compared to Sri Lanka (*P* < 0.001).

**Table 2.** Factors associated with uncontrolled hypertension in rural Bangladesh, Pakistan, and Sri Lanka

Characteristics	Basic models Odds ratio (95% CI) (*P*)	Multivariable model Odds ratio (95% CI) (*p*)
Demographic characteristics		
Educated (Ref: No formal education)	1.12 (0.86, 1.46) (0.385)	1.16 (0.86, 1.55) (0.335)
Working (Ref: Not working)	1.04 (0.79, 1.38) (0.784)	0.93 (0.68, 1.26) (0.637)
Not married (Ref: Married)	1.41 (1.10, 1.82) (0.007)	1.46 (1.10, 1.93) (0.008)
Anthropometric characteristics		
Obese/overweight (Ref: Nonobese)	1.19 (0.96, 1.47) (0.116)	1.11 (0.83, 1.49) (0.495)
Waist circumference (per 1 SD increase)	1.10 (0.99, 1.23) (0.071)	1.07 (0.92, 1.25) (0.397)
Life-style characteristics		
Current smoker (Ref: Nonsmoker)	0.99 (0.65, 1.50) (0.948)	1.00 (0.62, 1.59) (0.989)
Physical activity level (Ref: Inactive)	**(0.527)**	**(0.440)**
Minimally active	0.87 (0.68, 1.11) (0.261)	0.84 (0.64, 1.10) (0.207)
Highly active	0.92 (0.66, 1.28) (0.618)	0.91 (0.63, 1.30) (0.596)
Food-intake and 24-hrs urine sodium excretion		
Vegetables intake at least once per week (Ref: Less than once per week)	1.01 (0.62, 1.63) (0.980)	0.87 (0.52, 1.46) (0.589)
Fruits intake at least once per week (Ref: Less than once per week)	1.00 (0.81, 1.22) (0.983)	0.96 (0.77, 1.21) (0.756)
24-hrs sodium level (per 1 SD increase)	1.02 (0.91, 1.15) (0.675)	1.02 (0.90, 1.15) (0.807)
Socioeconomic characteristic		
International wealth index (per 1 SD decrease)	1.13 (1.00, 1.28) (0.045)	1.17 (1.02, 1.35) (0.028)
Self-reported health characteristics		
Chronic diseases related to hypertension (Ref.: Not reported)	0.68 (0.54, 0.84) (<0.001)	0.73 (0.57, 0.92) (0.009)
Heart disease (Ref: Not reported)	0.71 (0.54, 0.92) (0.010)	0.85 (0.63, 1.14) (0.282)
Stroke (Ref: Not reported)	0.84 (0.63, 1.12) (0.235)	0.88 (0.64, 1.20) (0.405)
Kidney disease (Ref: Not reported)	0.76 (0.45, 1.28) (0.299)	0.51 (0.28, 0.91) (0.023)
Diabetes (Ref.: Not reported)	1.20 (0.96, 1.51) (0.111)	1.06 (0.79, 1.43) (0.702)
Laboratory parameters		
Log_e_ urine spot albumin-to-creatinine ratio (per 1 SD increase)	1.40 (1.24, 1.57) (<0.001)	1.41 (1.24, 1.60) (<0.001)
Fasting plasma glucose level (per 1 SD increase)	1.26 (1.11, 1.42) (<0.001)	1.16 (0.99, 1.35) (0.068)
eGFR (per 1SD decrease)	1.20 (1.01, 1.43) (0.040)	1.23 (1.01, 1.49) (0.040)
HDL cholesterol level (per 1 SD increase)	1.12 (0.97, 1.28) (0.116)	1.04 (0.88, 1.22) (0.672)
LDL cholesterol level (per 1 SD increase)	1.22 (1.09, 1.36) (0.001)	1.14 (1.00, 1.30) (0.058)
Triglycerides level (per 1 SD increase)	1.05 (0.94, 1.17) (0.406)	0.97 (0.86, 1.10) (0.652)
Medication adherence		
Adherence level of antihypertensive medications (Ref: High)	**(<0.001)**	**(0.003)**
Medium	1.08 (0.84, 1.41) (0.544)	1.03 (0.77, 1.36) (0.862)
Low	1.54 (1.22, 1.95) (<0.001)	1.50 (1.16, 1.94) (0.002)
Currently on statins (Ref: Not currently on statin)	0.52 (0.39, 0.69) (<0.001)	0.62 (0.44, 0.87) (0.005)
Other characteristics		
Age (per 1 SD increase)	1.06 (0.96, 1.18) (0.268)	0.92 (0.78, 1.07) (0.275)
Male (Ref: Female)	0.84 (0.68, 1.04) (0.103)	1.06 (0.77, 1.46) (0.736)
Country (Ref: Sri Lanka)	**(<0.001)**	**(<0.001)**
Bangladesh	0.91 (0.62, 1.33) (0.637)	1.00 (0.61, 1.65) (0.994)
Pakistan	2.11 (1.38, 3.23) (0.001)	2.91 (1.60, 5.28) (<0.001)

Participant characteristics are defined in the methods section. Waist circumference, 24-hour sodium level, wealth index, Log urine spot albumin-to-creatinine ratio, fasting plasma glucose level, eGFR, HDL cholesterol level, LDL cholesterol level, triglycerides level, and age are presented as z-score standardized for mean and standard deviation.

*P*-values in bold represent joint-significance of all the categories using the Wald’s test. Each basic model was adjusted for country, age, and gender. Detailed description of the basic and multivariable models is provided in the methods section.

Abbreviations: CI, confidence interval; Ref, reference; eGFR, estimated glomerular filtration rate; HDL, high-density lipoprotein; LDL, low-density lipoprotein.

As shown in Table 2 column 3, in the multivariable model, the odds ratio (95% CI) of factors associated with uncontrolled BP were being single vs. married (1.46 (1.10, 1.93)), lower wealth index (1.17 (1.02, 1.35)), with vs. without self-reported kidney disease (0.51 (0.28, 0.91)), low adherence (1.50 (1.16, 1.94)), vs. high adherence to antihypertensive medications, receiving vs. not receiving statins (0.62 (0.44, 0.87)), high urine albumin-to-creatinine ratio (1.41 (1.24, 1.60)), low eGFR (1.23 (1.01, 1.49)) and living in Pakistan (2.91 (1.60, 5.28)) vs. Sri Lanka were independently and significantly associated with uncontrolled BP.

Statistically significant interactions were observed between Pakistan (reference to Sri Lanka) for several participant characteristics (education status, obese/overweight, waist circumference, fruit intake, HDL cholesterol, and triglycerides) in the multivariable model for uncontrolled BP (Supplementary Appendix Table 2). For example, the odds ratio of uncontrolled BP for obese/overweight was 3.34 times higher in Pakistan compared to Sri Lanka (*P* = 0.02). Similarly, the ratio of odds ratios (ROR) (interaction) for uncontrolled BP for every unit higher HDL cholesterol (mg/dl) was 2.07 (*P* ≤ 0.01) in Pakistan compared to Sri Lanka. Bangladesh differed statistically significantly for odds ratio of uncontrolled BP from Sri Lanka in education status, urine spot albumin-to-creatinine ratio, and adherence of antihypertensive medication., The odds ratio in Bangladesh were lower for log urine albumin-to-creatinine ratio (ROR = 0.65; *P* = 0.03), but higher for education (ROR = 3.36; *P* = 0.02) and medium adherence to antihypertensive medications (ROR = 1.99; *P* = 0.03) compared to Sri Lanka (Supplementary Appendix Table 2).

Sensitivity analysis yielded consistent results (Supplementary Appendix Tables 3–5).

## DISCUSSION

Using a common standardized protocol in 30 rural communities in South Asia, our study found that the majority (53%, 71%, and 57%) of individuals with treated hypertension have uncontrolled BP in Bangladesh, Pakistan, and Sri Lanka, respectively, with substantial cross-country variation. Our findings are unique in demonstrating the independent association of low within country wealth index, and low adherence to antihypertensive medicines with higher odds of uncontrolled BP in this rural region. These findings underscore the urgency for immediate public health action to target the identified barriers and enhance access to quality hypertension care services especially in the poorest of the poor populations living in the rural communities in South Asia.

Our findings are broadly consistent with other reports of a high proportion of adults with uncontrolled BP is consistent with studies in other low- and middle-income countries (LMICs). For example, 2 in 3 individuals on treatment for hypertension in 17 LMICs had uncontrolled BP in the PURE study.^23^ Poorer households have been associated with uncontrolled BP in India, Saudi Arabia, Malaysia, and Turkey^24^ Similar trends with low wealth and uncontrolled BP have been observed in Sub-Saharan Africa^25^ In part, much of the relationship between wealth inequalities in disparities in BP control is attributed to weak health systems resulting in poor access to medications especially in low income countries.^24^ Our study extends the literature by concomitantly accounting for lifestyle factors, comorbid conditions, and adherence to antihypertensive medications in the analysis. We demonstrate an independent relationship between low wealth index and higher odds of uncontrolled BP suggesting that factors in addition to health systems account for the observed association. Studies from the United Kingdom show that lower social positioning is associated with lower heart rate variability and autonomic dysfunction predisposing to higher BP and metabolic syndrome.^26^ Our findings highlight that the need of comprehensive pro-poor public health programs for hypertension management in rural South Asia.^27,28^

We found that low adherence to antihypertensive therapy was one of the key factors independently associated with high odds of uncontrolled BP in all 3 countries. Moreover, the majority of individuals in each of the 3 countries were receiving one antihypertensive medication only. The situation is similar in many LMICs. Recently reported China PEACE Million Persons Project showed the vast majority (81%) with poor BP control (70% of hypertensive population) were using one medication only.^29^ Epidemiological evidence from major clinical trials show that it takes 2–3 antihypertensive medications to control BP to <140/90 mmHg.^30^ In the United States, the overall hypertension control rate increased from 29% in NHANES 2001 to 2002 to 47% in NHANES 2009 to 2010. The proportion of patients taking multiple classes of antihypertensive medication increased from 37% to 48% during this period.^31^ Taken together, these findings indicate lack of effective titration of number of antihypertensive medications is likely to be a major barrier to hypertension control in rural South Asia.

Being single compared to married was associated with higher odds of uncontrolled BP in the multivariable model. High family support has also been associated with CVD benefits for both spouses.^32^ Strategies of engaging family members in hypertension management have shown promising results in rural China, must be encouraged in South Asia.^33^

While self-reported kidney disease was associated with better BP control, masked comorbidities identified by laboratory tests such as higher albuminuria and lower eGFR (both markers of kidney damage) were associated with higher odds of uncontrolled BP. Findings from the CRIC study indicate the majority of patients with CKD require 3 or more antihypertensive agents to control BP.^34^ Our findings underscore the importance of screening for CKD, as the vast majority are asymptomatic, to risk stratify individuals and BP treatment intensification among those with CKD in rural South Asian countries.

Finally, we observed that the proportion of individuals with uncontrolled BP was higher in Pakistan (about 2 in 3) than the other 2 countries (about 1 in 2). The number of antihypertensive medications used was lowest in Pakistan followed by Bangladesh. Unfortunately, there is no universal health coverage in Pakistan or Bangladesh, antihypertensive medications are not free in both the countries where more than 80% of expenditure on medications has been reported to be out-of-pocket, while selected generic antihypertensive medications based on the country essential medicines list of 2013 are provided free-of-cost in the public healthcare sector in Sri Lanka.^2^ These cross-country comparisons from the region highlight the need for reducing disparities in all of South Asia by ensuring access to quality antihypertensive medications with good coverage.^35^

Our study has some limitations. First, the clusters (communities) sampled in the 3 countries were not nationally representative. However, all communities (clusters) were randomly selected from the respective districts in the rural areas. Second, the cross-sectional design of the study does not establish causality of association with the determinants evaluated. However, the identified factors especially offer valuable information to be considered in designing targeted hypertension control programs. Third, only crude measures of dietary intake of fruit and vegetables were available. However, intake of produce is uniformly poor across the rural South Asian region including India.^36^ Fourth, MMAS-8 has been not validated in all the dialects of study populations. However, the scale underwent careful cycles of translation and back-translation, and pretesting prior to administration. Moreover, it has been validated in Urdu in Pakistan.^37^ Finally, 24-hour urine sodium was estimated from single spot urine sample rather than multiple 24-hour samples over several days. However, the latter is impractical especially in a field setting, and spot urine sodium has been shown to provide a reasonable albeit not perfect snapshot of dietary salt intake.^18^

Major strengths of our study are inclusion of representative rural community population with door to door sampling in 3 countries. The stratification of communities (clusters) by distance from government clinics and a minimum distance (7 kilometers) ensured representation of communities near and farther away from clinics in each country thereby enhancing the generalizability of findings to most rural areas in the 3 countries, and are consistent with other reports of poor hypertension control in rural South Asia.^24^ The standardized protocols for BP measurements in all 3 countries, using AAMI and BHS recommended digital Omron digital device, and multiple readings of BP, and use of Morisky scale for adherence enhance the validity of our findings. Thus, we believe our findings are robust.

It is also important to underscore that Bangladesh, Pakistan, and Sri Lanka, each have a government-funded community health workers’ (CHW) program embedded in their primary health system for basic maternal and child care and family planning services.^2^ However, this cadre of CHW does not have the mandate (or training) to provide noncommunicable disease or hypertension screening or related education.^38^ Our previous work in urban Pakistan suggests that a combined strategy of training CHW coupled with training providers in updated hypertension management is effective and cost-effective in lowering BP.^39^ The ongoing COBRA-BPS trial in rural Bangladesh, Pakistan, and Sri Lanka is evaluating these interventions with additional components including a financing model for integration in the rural public health infrastructure.^10^

In conclusion, our study highlights that the majority of individuals with hypertension in rural communities in Bangladesh, Pakistan, and Sri Lanka have uncontrolled BP, and there are cross-country as well as within country disparities in BP control in the region. The odds for uncontrolled hypertension were especially high among individuals residing in Pakistan where number of antihypertensive medications used was the lowest. In all the 3 countries, the odds of uncontrolled hypertension were higher in individuals who were single vs. married, residing in lower wealth index households, with lower adherence to antihypertensive medications, and in the presence of albuminuria or reduced kidney function. Our findings have significant public health implications and underscore the urgency for prioritizing comprehensive hypertension management program in the rural communities in South Asia.

## SUPPLEMENTARY MATERIAL

Supplementary data are available at *American Journal of Hypertension* online.

Supplementary Appendix Figure

Supplementary Appendix Table

## DISCLOSURE

The authors declared no conflict of interest.
