# Supplementary material for: Determinants of Uncontrolled Hypertension in Rural Communities in South Asia—Bangladesh, Pakistan, and Sri Lanka
Source: Am J Hypertens. 2018 Apr 26;31(11):1205–14. doi: 10.1093/ajh/hpy071 (PMC6188532; doi:10.1093/ajh/hpy071)
Supplement: Supplementary Appendix Figure [file hpy071_suppl_supplementary_appendix_figure.docx]

**Appendix Figure 1. Enrollment Flow Chart – 30 communities in Bangladesh, Pakistan, Sri Lanka.**

Screened (n=11510, aged 40+)

30 clusters

No hypertension (n=7878, 68.4%)

Severe physical or mental illness or pregnancy (65, 0.6%)

Incomplete information (n=655, 5.7%)

Eligible (n=2912, aged 40+ with hypertension)

30 clusters

Drop-out before baseline visit (n=181, 6.2%)

Uncontactable (n=63, 2.2%)

Adverse event (n=1, 0.0%)

Death (n=1, 0.0%)

Withdrew consent (n=87, 3.0%)

Sickness/Pregnancy (n=21, 0.7%)

Others (n=8, 0.3%)

Reached quota for target enrollment thus excluded (n=88, 3.0%)

Enrolled (n=2643, aged 40+ with hypertension)

30 Clusters
