# Supplementary material for: Determinants of Uncontrolled Hypertension in Rural Communities in South Asia—Bangladesh, Pakistan, and Sri Lanka
Source: Am J Hypertens. 2018 Apr 26;31(11):1205–14. doi: 10.1093/ajh/hpy071 (PMC6188532; doi:10.1093/ajh/hpy071)
Supplement: Supplementary Appendix Table [file hpy071_suppl_supplementary_appendix_table.docx]

**Appendix Table 1. Characteristics of individuals with treated hypertension by hypertension status in rural Bangladesh, Pakistan, and Sri Lanka.**

|  | **Bangladesh (N=670)** | | | **Pakistan (N=361)** | | | **Sri Lanka (N=687)** | | |
| --- | --- | --- | --- | --- | --- | --- | --- | --- | --- |
| **Characteristics** | **Uncontrolled hypertensive patients* (N=354, 52.8%)** | **Controlled hypertensive patients (N=316, 47.2%)** | ***p*** | **Uncontrolled hypertensive patients* (N=255, 70.6%)** | **Controlled hypertensive patients (N=106, 29.4%)** | ***p*** | **Uncontrolled hypertensive patients* (N=388, 56.5%)** | **Controlled hypertensive patients (N=299, 43.5%)** | ***p*** |
| Demographic characteristics |  |  |  |  |  |  |  |  |  |
| Age (years), Mean (SD) | 57.43 (11.14) | 56.31 (11.15) | 0.20 | 58.18 (11.96) | 55.35 (11.78) | 0.04 | 63.14 (10.59) | 64.38 (10.23) | 0.12 |
| Male, n (%) | 107 (30.2) | 108 (34.2) | 0.27 | 75 (29.4) | 31 (29.2) | 0.98 | 105 (27.1) | 97 (32.4) | 0.13 |
| Education, n (%) |  |  | 0.52 |  |  | 0.95 |  |  | 0.22 |
| No formal education | 166 (46.9) | 156 (49.4) |  | 170 (66.7) | 71 (67.0) |  | 19 (4.9) | 9 (3.0) |  |
| Formal education | 188 (53.1) | 160 (50.6) |  | 85 (33.3) | 35 (33.0) |  | 369 (95.1) | 290 (97.0) |  |
| Currently working, n (%) | 77 (21.8) | 84 (26.6) | 0.14 | 65 (25.5) | 29 (27.4) | 0.71 | 96 (24.7) | 70 (23.4) | 0.69 |
| Married, n (%) | 253 (71.5) | 257 (81.3) | <0.01 | 172 (67.5) | 79 (74.5) | 0.18 | 237 (61.1) | 199 (66.6) | 0.14 |
| Anthropometric characteristics |  |  |  |  |  |  |  |  |  |
| Obese/overweight [≥23.5 BMI], n (%) | 199 (56.2) | 172 (54.4) | 0.57 | 178 (69.8) | 67 (63.2) | 0.25 | 233 (60.1) | 165 (55.2) | 0.32 |
| BMI (kg/m^2^), Mean (SD) | 24.40 (3.93) | 24.17 (3.87) | 0.44 | 26.46 (5.93) | 26.50 (6.15) | 0.96 | 25.20 (4.75) | 24.67 (4.73) | 0.16 |
| Waist circumference (cm), Mean (SD) | 86.38 (10.67) | 86.22 (10.77) | 0.85 | 91.73 (14.46) | 92.68 (17.01) | 0.59 | 90.10 (12.09) | 88.12 (13.07) | 0.04 |
| Socio-economic characteristics |  |  |  |  |  |  |  |  |  |
| International wealth index, Mean (SD) | 50.01 (17.07) | 52.28 (17.56) | 0.09 | 58.68 (19.95) | 61.12 (17.19) | 0.27 | 73.01 (17.18) | 74.38 (16.48) | 0.29 |
| Life-style characteristics |  |  |  |  |  |  |  |  |  |
| Currently smoking, n (%) | 25 (7.1) | 31 (9.8) | 0.20 | 27 (10.6) | 9 (8.5) | 0.55 | 13 (3.4) | 10 (3.3) | >0.99 |
| Physical activity level, n (%) |  |  | 0.19 |  |  | 0.17 |  |  | 0.31 |
| Inactive | 70 (19.8) | 58 (18.4) |  | 109 (42.7) | 35 (33.0) |  | 111 (28.6) | 75 (25.1) |  |
| Minimally active | 241 (68.1) | 204 (64.6) |  | 100 (39.2) | 52 (49.1) |  | 203 (52.3) | 174 (58.2) |  |
| Highly active | 43 (12.1) | 54 (17.1) |  | 46 (18.0) | 19 (17.9) |  | 74 (19.1) | 50 (16.7) |  |
| Self-reported health characteristics |  |  |  |  |  |  |  |  |  |
| Chronic diseases related to hypertension, n (%) | 119 (33.6) | 122 (38.6) | 0.18 | 42 (16.5) | 36 (34.0) | <0.001 | 89 (22.9) | 91 (30.4) | 0.03 |
| Heart disease, n (%) | 61 (17.2) | 62 (19.6) | 0.43 | 23 (9.0) | 18 (17.0) | 0.03 | 55 (14.2) | 61 (20.4) | 0.03 |
| Stroke, n (%) | 72 (20.3) | 69 (21.8) | 0.64 | 23 (9.0) | 14 (13.2) | 0.23 | 33 (8.5) | 33 (11.0) | 0.26 |
| Kidney disease, n (%) | 9 (2.5) | 16 (5.1) | 0.09 | 7 (2.7) | 5 (4.7) | 0.34 | 17 (4.4) | 10 (3.3) | 0.49 |
| Diabetes, n (%) | 86 (24.3) | 79 (25.0) | 0.83 | 43 (16.9) | 15 (14.2) | 0.52 | 145 (37.4) | 91 (30.4) | 0.06 |
| Morisky adherence level for anti-hypertensive medications, n (%) |  |  | 0.08 |  |  | 0.06 |  |  | <0.01 |
| Low | 161 (45.5) | 128 (40.5) |  | 123 (48.2) | 37 (34.9) |  | 167 (43.0) | 93 (31.1) |  |
| Medium | 97 (27.4) | 77 (24.4) |  | 50 (19.6) | 28 (26.4) |  | 87 (22.4) | 89 (29.8) |  |
| High | 96 (27.1) | 111 (35.1) |  | 82 (32.2) | 41 (38.7) |  | 134 (34.5) | 117 (39.1) |  |
| Number of anti-hypertensive medications, n (%) |  |  | 0.04 |  |  | 0.10 |  |  | 0.11 |
| 1 | 253 (71.5) | 198 (62.7) |  | 224 (87.8) | 85 (80.2) |  | 208 (53.6) | 184 (61.5) |  |
| 2 | 83 (23.4) | 101 (32.0) |  | 30 (11.8) | 19 (17.9) |  | 129 (33.2) | 84 (28.1) |  |
| ≥3 | 18 (5.1) | 17 (5.4) |  | 1 (0.4) | 2 (1.9) |  | 51 (13.1) | 31 (10.4) |  |
| Currently on statins, n (%) | 9 (2.5) | 14 (4.4) | 0.18 | 9 (3.5) | 9 (8.5) | 0.049 | 136 (35.1) | 147 (49.2) | <0.001 |
| Food-intake and related laboratory measures |  |  |  |  |  |  |  |  |  |
| Vegetables intake (at least once a week), n (%) | 351 (99.2) | 306 (96.8) | 0.03 | 252 (98.8) | 105 (99.1) | 0.85 | 349 (89.9) | 273 (91.3) | 0.55 |
| Fruits intake (at least once a week), n (%) | 204 (57.6) | 201 (63.6) | 0.11 | 136 (53.3) | 44 (41.5) | 0.04 | 200 (51.5) | 154 (51.5) | >0.99 |
| 24-hrs urine sodium (g/day), Mean (SD) | 4.47 (1.59) | 4.46 (1.47) | 0.94 | 4.20 (1.46) | 4.17 (1.47) | 0.88 | 5.26 (1.34) | 5.13 (1.40) | 0.21 |
| Log_e_ urine spot albumin-to-creatinine ratio (mg/g), Mean (SD) | 2.85 (1.74) | 2.31 (1.60) | <0.001 | 2.56 (1.43) | 2.13 (1.06) | 0.01 | 3.26 (0.96) | 2.95 (0.75) | <0.001 |
| Systolic blood pressure (mmHg), Mean (SD) | 154.60 (16.13) | 122.78 (10.51) | <0.001 | 160.69 (20.00) | 122.12 (11.05) | <0.001 | 160.69 (18.58) | 124.65 (10.78) | <0.001 |
| Diastolic blood pressure (mmHg), Mean (SD) | 94.24 (10.95) | 75.37 (8.84) | <0.001 | 96.37 (13.97) | 74.00 (8.95) | <0.001 | 93.64 (12.31) | 74.16 (9.28) | <0.001 |
| Fasting plasma glucose (mg/dL), Mean (SD) | 113.59 (44.73) | 111.26 (40.66) | 0.49 | 120.74 (62.01) | 103.23 (35.11) | 0.01 | 127.45 (52.08) | 114.89 (30.72) | <0.001 |
| eGFR (mL/min per 1.73 m^2^), Mean (SD) | 77.60 (19.45) | 79.79 (19.79) | 0.15 | 94.66 (20.19) | 96.52 (24.34) | 0.49 | 50.74 (12.85) | 51.86 (13.03) | 0.28 |
| Total cholesterol (mg/dL), Mean (SD) | 199.79 (48.06) | 194.27 (41.25) | 0.12 | 180.41 (41.72) | 162.65 (42.31) | <0.01 | 210.86 (49.59) | 197.42 (47.33) | <0.01 |
| HDL cholesterol (mg/dL), Mean (SD) | 38.34 (11.04) | 38.39 (10.17) | 0.95 | 43.46 (11.95) | 38.51 (10.56) | <0.01 | 55.33 (9.20) | 54.71 (9.87) | 0.41 |
| LDL cholesterol (mg/dL), Mean (SD) | 134.62 (39.24) | 131.61 (35.80) | 0.31 | 112.14 (34.72) | 101.53 (33.90) | 0.01 | 129.96 (42.06) | 119.57 (40.36) | <0.01 |
| Triglycerides (mg/dL), Mean (SD) | 172.84 (99.04) | 174.24 (103.13) | 0.86 | 165.49 (105.35) | 166.65 (113.49) | 0.93 | 127.73 (67.63) | 115.89 (54.89) | 0.02 |

* Uncontrolled hypertension was defined as systolic blood pressure ≥140 mmHg or diastolic blood pressure ≥90 mmHg. Participant characteristics are defined in the methods section.

SD Standard deviation; BMI Body mass index; eGFR Estimated glomerular filtration rate; HDL High-density lipoprotein; LDL Low-density lipoprotein.

**Appendix Table 2. Summary of multivariable model for uncontrolled hypertension including interaction between country and risk factors of those who took anti-hypertensive medications.**

| **Characteristics** | **Multivariable model**  **Odds ratio (95% CI) [*p*]** |
| --- | --- |
| Demographic characteristics |  |
| Educated (Ref: No formal education) | 0.42 (0.16, 1.10) [0.08] |
| Bangladesh × Educated | 3.36 (1.20, 9.42) [0.02] |
| Pakistan × Educated | 3.91 (1.14, 13.42) [0.03] |
| Working (Ref: Not working) | 1.16 (0.75, 1.81) [0.50] |
| Bangladesh × Working | 0.69 (0.37, 1.28) [0.24] |
| Pakistan × Working | 0.88 (0.37, 2.07) [0.77] |
| Not married (Ref: Married) | 1.36 (0.91, 2.04) [0.14] |
| Bangladesh × Not married | 1.26 (0.71, 2.27) [0.43] |
| Pakistan × Not married | 1.12 (0.47, 2.66) [0.80] |
| Anthropometric characteristics |  |
| Obese/overweight (Ref: Non-obese) | 0.84 (0.53, 1.34) [0.46] |
| Bangladesh × Obese/overweight | 1.40 (0.73, 2.66) [0.31] |
| Pakistan × Obese/overweight | 3.34 (1.24, 8.99) [0.02] |
| Waist circumference (per 1 SD increase) | 1.28 (1.01, 1.62) [0.04] |
| Bangladesh × Waist circumference | 0.77 (0.53, 1.12) [0.17] |
| Pakistan × Waist circumference | 0.59 (0.38, 0.91) [0.02] |
| Life-style characteristics |  |
| Current smoker (Ref: Non-smoker) | 0.99 (0.36, 2.74) [0.98] |
| Bangladesh × Current smoker | 0.79 (0.24, 2.63) [0.71] |
| Pakistan × Current smoker | 1.79 (0.40, 7.98) [0.45] |
| Physical activity level (Ref: Inactive) |  |
| Minimally active | 0.72 (0.47, 1.10) [0.13] |
| Highly active | 1.10 (0.63, 1.90) [0.74] |
| Bangladesh × Minimally active | 1.55 (0.84, 2.88) [0.16] |
| Bangladesh × Highly active | 0.81 (0.35, 1.87) [0.62] |
| Pakistan × Minimally active | 0.76 (0.34, 1.73) [0.52] |
| Pakistan × Highly active | 0.66 (0.23, 1.92) [0.45] |
| Food-intake and urine sodium excretion |  |
| Vegetables intake at least once per week (Ref: Less than once per week) | 0.85 (0.46, 1.57) [0.61] |
| Bangladesh × Vegetable intake | 0.37 (0.08, 1.69) [0.20] |
| Pakistan × Vegetable intake | 1.00 (Not estimable) |
| Fruits intake at least once per week (Ref: Less than once per week) | 1.01 (0.71, 1.45) [0.95] |
| Bangladesh × Fruits intake | 1.26 (0.75, 2.12) [0.38] |
| Pakistan × Fruits intake | 0.46 (0.22, 0.98) [0.04] |
| 24-hrs sodium (per 1 SD increase) | 0.98 (0.79, 1.21) [0.86] |
| Bangladesh × 24-hrs sodium | 1.03 (0.78, 1.37) [0.83] |
| Pakistan × 24-hrs sodium | 1.00 (0.67, 1.49) [>0.99] |
| Socioeconomic characteristics |  |
| International wealth index (per 1 SD decrease) | 1.19 (0.94, 1.52) [0.15] |
| Bangladesh × International wealth index | 0.97 (0.69, 1.35) [0.86] |
| Pakistan × International wealth index | 1.08 (0.68, 1.42) [0.75] |
| Self-reported health characteristics |  |
| Chronic diseases related to hypertension (Ref.: Not reported) | 0.74 (0.49, 1.13) [0.17] |
| Bangladesh × Chronic disease related to hypertension | 1.08 (0.63, 1.88) [0.77] |
| Pakistan × Chronic disease related to hypertension | 0.52 (0.23, 1.18) [0.12] |
| Heart disease (Ref: No reported) | 0.74 (0.45, 1.19) [0.21] |
| Bangladesh × Heart disease | 1.31 (0.68, 2.53) [0.43] |
| Pakistan × Heart disease | 0.82 (0.30, 2.26) [0.70] |
| Stroke (Ref: Not reported) | 0.92 (0.50, 1.70) [0.80] |
| Bangladesh × Stroke | 0.96 (0.46, 2.01) [0.92] |
| Pakistan × Stroke | 0.73 (0.23, 2.34) [0.59] |
| Kidney disease (Ref: No reported) | 0.77 (0.28, 2.12) [0.61] |
| Bangladesh × Kidney disease | 0.45 (0.11, 1.78) [0.26] |
| Pakistan × Kidney disease | 0.34 (0.05, 2.28) [0.27] |
| Diabetes (Ref: Not reported) | 1.09 (0.70, 1.70) [0.69] |
| Bangladesh × Diabetes | 0.85 (0.44, 1.63) [0.62] |
| Pakistan × Diabetes | 1.12 (0.36, 3.52) [0.85] |
| Laboratory parameters |  |
| Log_e_ urine spot ACR (per 1 SD increase) | 2.08 (1.43, 3.03) [<0.001] |
| Bangladesh × Log urine spot ACR | 0.65 (0.43, 0.97) [0.03] |
| Pakistan × Log urine spot ACR | 0.74 (0.43, 1.27) [0.27] |
| Fasting plasma glucose (per 1 SD increase) | 1.24 (0.95, 1.61) [0.11] |
| Bangladesh × Fasting plasma glucose | 0.78 (0.54, 1.11) [0.16] |
| Pakistan × Fasting plasma glucose | 1.23 (0.71, 2.15) [0.46] |
| eGFR (per 1 SD decrease) | 1.00 (0.68, 1.49) [0.98] |
| Bangladesh × eGFR | 1.20 (0.78, 1.85) [0.41] |
| Pakistan × eGFR | 1.43 (0.82, 2.44) [0.21] |
| HDL cholesterol level (per 1 SD increase) | 0.88 (0.66, 1.16) [0.36] |
| Bangladesh × HDL | 1.08 (0.75, 1.56) [0.67] |
| Pakistan × HDL | 2.07 (1.21, 3.54) [<0.01] |
| LDL cholesterol (per 1 SD increase) | 1.19 (0.96, 1.47) [0.12] |
| Bangladesh × LDL | 0.93 (0.70, 1.25) [0.64] |
| Pakistan × LDL | 1.06 (0.67, 1.68) [0.80] |
| Triglycerides (per 1 SD increase) | 1.29 (0.96, 1.73) [0.09] |
| Bangladesh × Triglycerides | 0.72 (0.51, 1.01) [0.06] |
| Pakistan × Triglycerides | 0.66 (0.99, 1.95) [0.04] |
| Medication adherence |  |
| Adherence level of antihypertensive medication (Ref: High) |  |
| Medium | 0.73 (0.47, 1.14) [0.16] |
| Low | 1.54 (1.01, 2.34) [0.04] |
| Bangladesh × Medium | 1.99 (1.05, 3.76) [0.03] |
| Bangladesh × Low | 0.91 (0.51, 1.64) [0.76] |
| Pakistan × Medium | 1.05 (0.41, 2.69) [0.92] |
| Pakistan × Low | 1.22 (0.53, 2.80) [0.65] |
| Currently on statins (Ref: Not currently on statin) | 0.55 (0.36, 0.83) [<0.01] |
| Bangladesh × Currently on statins | 1.38 (0.47, 4.08) [0.56] |
| Pakistan × Currently on statins | 1.19 (0.28, 5.02) [0.82] |
| Other characteristics |  |
| Age (per 1 SD increase) | 0.92 (0.78, 1.08) [0.32] |
| Male (Ref: Female) | 1.05 (0.74, 1.49) [0.78] |
| Country (Ref: Sri Lanka) |  |
| Bangladesh | 0.12 (0.03, 0.51) [<0.01] |
| Pakistan | 0.86 (0.16, 4.49) [0.86] |

CI Confidence interval; Ref Reference; ACR albumin-to-creatinine ratio; eGFR Estimated glomerular filtration rate; HDL High-density lipoprotein; LDL Low-density lipoprotein.

Participant characteristics are defined in the methods section. Waist circumference, 24-hrs sodium level, international wealth index, Log urine spot albumin-to-creatinine ratio, fasting plasma glucose level, eGFR, HDL cholesterol level, LDL cholesterol level, triglycerides level, and age are presented as z-score standardized for mean and standard deviation.

**Appendix Table 3. Proportion with uncontrolled blood pressure among those with treated hypertension by cluster distance.**

| **Cluster distance** | **Number of uncontrolled hypertensive patients/Cluster size (%)** | | |
| --- | --- | --- | --- |
|  | **Bangladesh** | **Pakistan** | **Sri Lanka** |
| Far | 148/270 (54.8) | 114/155 (73.5) | 155/282 (55.0) |
| Near | 206/400 (51.5) | 141/206 (68.4) | 233/405 (57.5) |

**Appendix Table 4. Proportion with uncontrolled blood pressure among those with treated hypertension by self-reported chronic disease groups.**

| ***** Self reported chronic diseases related to hypertension | **All counties (N = 1718)** | | **Bangladesh (N = 670)** | | **Pakistan (N = 361)** | | **Sri Lanka (N = 687)** | |
| --- | --- | --- | --- | --- | --- | --- | --- | --- |
|  | **Low adherence of antihypertensive medication, n (%)** | **Uncontrolled hypertensive patients, n (%)** | **Low adherence of antihypertensive medication, n (%)** | **Uncontrolled hypertensive patients, n (%)** | **Low adherence of antihypertensive medication, n (%)** | **Uncontrolled hypertensive patients, n (%)** | **Low adherence of antihypertensive medication, n (%)** | **Uncontrolled hypertensive patients, n (%)** |
| Without chronic disease | 517 (30.1) | 747 (43.5) | 188 (28.1) | 235 (35.1) | 133 (36.8) | 213 (59.0) | 196 (28.5) | 299 (43.5) |
| With chronic disease | 192 (11.2) | 250 (14.6) | 101 (15.1) | 119 (17.8) | 27 (7.5) | 42 (11.6) | 64 (9.3) | 89 (13.0) |

chronic disease related to hypertension were defined as self reported presence of heart disease, stroke, or kidney disease.

**Appendix Table 5. Proportion of with uncontrolled blood pressure among those with treated hypertension by economic status.**

| **Economic status based on international wealth index** | **All counties (N = 1718)** | | **Bangladesh (N = 670)** | | **Pakistan (N = 361)** | | **Sri Lanka (N = 687)** | |
| --- | --- | --- | --- | --- | --- | --- | --- | --- |
|  | **Low adherence of antihypertensive medication, n (%)** | **Uncontrolled hypertensive patients, n (%)** | **Low adherence of antihypertensive medication, n (%)** | **Uncontrolled hypertensive patients, n (%)** | **Low adherence of antihypertensive medication, n (%)** | **Uncontrolled hypertensive patients, n (%)** | **Low adherence of antihypertensive medication, n (%)** | **Uncontrolled hypertensive patients, n (%)** |
| Poor [< 15 percentile] | 110 (43.1) | 159 (62.4) | 43 (43.9) | 53 (54.1) | 31 (54.4) | 46 (80.7) | 36 (36.0) | 60 (60.0) |
| Middle [15 – 85 percentile] | 498 (42.3) | 683 (58.0) | 199 (43.8) | 246 (54.2) | 112 (46.7) | 165 (68.8) | 187 (38.7) | 272 (56.3) |
| Rich [>85 percentile] | 99 (35.0) | 154 (54.4) | 47 (39.8) | 55 (46.6) | 16 (25.8) | 43 (69.4) | 36 (35.0) | 56 (54.4) |
